# Differential miRNA Expression in the Liver of Balb/c Mice Protected by Vaccination during Crisis of *Plasmodium chabaudi* Blood-Stage Malaria

**DOI:** 10.3389/fmicb.2016.02155

**Published:** 2017-01-11

**Authors:** Mohamed A. Dkhil, Saleh A. Al-Quraishy, Abdel-Azeem S. Abdel-Baki, Denis Delic, Frank Wunderlich

**Affiliations:** ^1^Department of Zoology, College of Science, King Saud UniversityRiyadh, Saudi Arabia; ^2^Department of Zoology and Entomology, Faculty of Science, Helwan UniversityCairo, Egypt; ^3^Department of Zoology, Faculty of Science, Beni-Suef UniversityBeni-Suef, Egypt; ^4^Boehringer-Ingelheim PharmaBiberach, Germany; ^5^Department of Biology, Heinrich-Heine-UniversityDuesseldorf, Germany

**Keywords:** miRNA, liver, blood-stage malaria, *Plasmodium chabaudi*, protective vaccination

## Abstract

MicroRNAs are increasingly recognized as epigenetic regulators for outcome of diverse infectious diseases and vaccination efficacy, but little information referring to this exists for malaria. This study investigates possible effects of both protective vaccination and *P. chabaudi* malaria on the miRNome of the liver as an effector against blood-stage malaria using miRNA microarrays and quantitative PCR. *Plasmodium chabaudi* blood-stage malaria takes a lethal outcome in female Balb/c mice, but a self-healing course after immunization with a non-infectious blood-stage vaccine. The liver robustly expresses 71 miRNA species at varying levels, among which 65 miRNA species respond to malaria evidenced as steadily increasing or decreasing expressions reaching highest or lowest levels toward the end of the crisis phase on day 11 *p.i*. in lethal malaria. Protective vaccination does not affect constitutive miRNA expression, but leads to significant (*p* < 0.05) changes in the expression of 41 miRNA species, however evidenced only during crisis. In vaccination-induced self-healing infections, 18 miRNA-species are up- and 14 miRNA-species are down-regulated by more than 50% during crisis in relation to non-vaccinated mice. Vaccination-induced self-healing and survival of otherwise lethal infections of *P. chabaudi* activate epigenetic miRNA-regulated remodeling processes in the liver manifesting themselves during crisis. Especially, liver regeneration is accelerated as suggested by upregulation of let-7a-5p, let-7b-5p, let-7c-5p, let-7d-5p, let-7f-5p, let-7g-5p, let-7i-5p, miR-26a, miR-122-5p, miR30a, miR27a, and mir-29a, whereas the up-regulated expression of miR-142-3p by more than 100% is compatible with the view of enhanced hepatic erythropoiesis, possibly at expense of megakaryopoiesis, during crisis of *P. chabaudi* blood-stage malaria.

## Introduction

The World Health Organization has reported about 214 million new cases of malaria with about 438,000 deaths worldwide in 2015 (WHO, 2015). A vaccine for an effective and reliable anti-malaria prophylaxis is not yet available, despite enormous efforts during the last 35 years (Halbroth and Draper, 2015; Hoffman et al., 2015; Miura, 2016). Until recently, the RTS,S/AS01 has been regarded as the most advanced vaccine candidate. Indeed, the European Medicines Agency has approved RTS,S/AS01 for immunizations of very young children, however the WHO has not recommended its inclusion in the Expanded Programme of Immunizations (Birkitt, 2016; Gosling and von Seidlein, 2016). This points to the urgency of more basic research to uncover those mechanisms of the host defense, which a vaccine has to activate to provide protection against malaria.

The experimental malaria *P. chabaudi* in mice shares several characteristics with *P. falciparum*, the most dangerous malaria species for humans (Longley et al., 2011; Stephens et al., 2012). It is a convenient model to study blood-stage malaria. The outcome of *P. chabaudi* blood-stage malaria is controlled by genes of the mouse-MHC, the H-2 complex, genes of the non-H-2 background, and environmental factors such as testosterone (Wunderlich et al., 1988d). Moreover, there is a vaccination technique available by which survival of mice, being naturally susceptible to malaria, can be raised from 0% to over 80% (Wunderlich et al., 1988a; Krücken et al., 2009). This technique, which uses a non-infectious vaccine consisting of surface membranes isolated from *P. chabaudi*-infected erythrocytes containing parasite-synthesized proteins (Wunderlich et al., 1988b,c; Fontaine et al., 2012), converts lethal blood-stage infections to take a self-healing course (Krücken et al., 2009). Upon challenging with 10^6^ *P. chabaudi*-parasitized erythrocytes, all non-vaccinated mice succumb to malaria during the crisis phase of infection, whereas the majority of vaccinated mice survive the infections and generate long-lasting immune mechanisms against homologous re-challenge (Krücken et al., 2009).

The spleen is currently envisioned as the key effector organ against blood-stage malaria (Engwerda et al., 2005; Del Portillo et al., 2012). Indeed, the spleen is equipped with a unique system to eliminate senescent and aberrant erythrocytes including *P*.-infected erythrocytes. However, there is accumulating evidence that, also the liver, in particular the erythrophagocytotic Kupffer cells are able to remove senescent and other aberrant erythrocytes including *P*.-infected erythrocytes (Terpstra and van Berkel, 2000; Otogata et al., 2007; Delic et al., 2010; Lee et al., 2011; Wunderlich et al., 2014). In self-healing infections of *P. chabaudi*, it has been described that the liver dramatically increases its uptake of injected particles at peak parasitemia and during subsequent crisis, whereas, concomitantly, the spleen dramatically declines its uptake of injected particulate material including fluorescently labeled *P. chabaudi*-infected erythrocytes (Krücken et al., 2005, 2009). It is therefore rather likely that the liver, rather than the spleen, massively removes *P. chabaudi*-infected erythrocytes from circulation during crisis in vaccination-induced self-healing infections of *P. chabaudi* (Wunderlich et al., 2014). Very recently, the liver has been even described as the primary organ for a unique mechanism of rapid on-demand erythrocyte disposal (Theurl et al., 2016).

Current evidence demonstrates that miRNAs are critical for maintaining phenotype and functionality of the liver (Chen and Verfaillie, 2014), and that diverse diseases of the liver are associated with dysregulations in the miRNA signature (Szabo and Bala, 2013; Bandiera et al., 2015). MicroRNAs are non-coding single-stranded RNAs of 22 nts in length, which posttranscriptionally regulate gene expression by affecting both stability and translation of mRNAs. At least one third of human genes are estimated to be targeted by miRNAs (He and Hannon, 2004). MicroRNAs are involved in regulating epigenetic networks within cells and via exosomes between cells (Vyas and Dhawan, 2016). They are increasingly recognized as to be critical for the outcome of diverse infectious diseases (Bettencourt et al., 2016; Das et al., 2016; Verma et al., 2016). Also, miRNAs appear to be critical for efficacy of vaccination (Corral-Fernández et al., 2016; Shim et al., 2016; Wang et al., 2016). However, there is still little information available about host miRNAs in malaria, especially with respect to course and outcome of malaria infections and vaccination efficacy (Cohen et al., 2015; Rubio et al., 2016). At least, it is known that miRNAs of the liver are responsive to blood-stage infections of *P. chabaudi* malaria (Delic et al., 2011). The aim of this paper is therefore to analyze as to whether protective vaccination against *P. chabaudi* malaria leads to changes in the overall expression of miRNAs in the liver during self-healing infections of *P. chabaudi* in comparison to lethal infections in non-vaccinated female Balb/c mice.

## Materials and methods

### Mice

Balb/c mice were bred under specified pathogen-free conditions. For the experiments, only female Balb/c mice, approximately 10–14 weeks old, were used. They were housed in plastic cages, received a standard diet (Woehrlin, Bad Salzuflen, Germany) and water *ad libitum*. This study was carried out in strict accordance with the German law on animal protection. The keeping of mice and the experimental protocol of the study were officially approved by the State-controlled Committee on the Ethics of Animal Experiments and were regularly controlled, without being previously announced, by the local authorities. All efforts were made to minimize suffering of mice.

### *P. chabaudi* malaria

A non-clonal line of *P. chabaudi* is used in our laboratory since 1982 (Wunderlich et al., 1982), which resembles to *P. chabaudi chabaudi AS* as described previously (Wunderlich et al., 2005; Krücken et al., 2009). Blood-stage infections were routinely maintained in outbred mice under sterile conditions by weekly passages of infected blood. Balb/c mice were *i.p*. challenged with 10^6^ *P. chabaudi*-infected erythrocytes. Erythrocytes were counted in a Neubauer chamber and parasitemia was evaluated in Giemsa-stained smears from tail blood.

### Vaccination

Mice were vaccinated as performed previously (Krücken et al., 2009). As a vaccine we have used host cell plasma membranes, isolated in the form of ghosts from *P. chabaudi*-parasitized red blood cells as described elsewhere (Wunderlich et al., 1985, 1987). Approximately 10^6^ ghosts suspended in 100 μl Freund's complete adjuvant were injected on week 3 and week 1 before challenge with 10^6^ *P. chabaudi*-parasitized erythrocytes (Krücken et al., 2009). Controls received only the adjuvant.

### RNA isolation

Livers were removed from sacrificed mice, rapidly frozen in liquid nitrogen and stored at −80°C until use. Frozen livers were grounded in a mortar under liquid nirogen and aliquots were used to isolate total RNA using Trizol (Sigma-Aldrich). An additional cleaning step was performed by using the miRNeasy Kit (Qiagen). RNA quality and integrity were determined using the Agilent RNA 6000 Nano Kit on the Agilent 2100 Bioanalyzer platform (Agilent Technologies). RNA was quantified by measuring A260 nm on the ND-1000 Spectrophotometer (NanoDrop Technologies).

### Labeling of miRNA

Labeling of the samples was performed as detailed in the “miRNA Microarray System with miRNA Complete Labeling and Hyb Kit” protocol (version 2.4, part number G4170-90011). In brief, 100 ng of each total RNA sample was used for the labeling step using the miRNA Complete Labeling and Hyb Kit (Agilent Technologies).

### Hybridization of agilent microRNA microarrays

The used Agilent Mouse microRNA Microarrays 8 × 60 K v19 (Design ID 046065) contain 8 arrays per slide and one array is equipped with probes for detecting 1247 mouse miRNAs. Hybridization was performed according to the “miRNA Complete Labeling and Hyb Kit” protocol (version 2.4, part number G4170-90011) using the miRNA Complete Labeling and Hyb Kit (Agilent Technologies). In brief, Cy3-labeled RNA in hybridization buffer was hybridized overnight (20 h, 55°C) to the microarrays using Agilent's recommended hybridization chamber and oven. Thereafter, the microarrays were washed once with the Agilent Gene Expression Wash Buffer 1 for 5 min at room temperature and once by a second wash with preheated Agilent Gene Expression Wash Buffer 2 (37°C) for 5 min.

### Scanning and data analysis

Agilent's Microarray Scanner System G2505C (Agilent Technologies) was used to detect fluorescence signals of the hybridized Agilent Microarrays. The Agilent Feature Extraction Software (FES) 10.7.3.1 was used to read out and process the microarray image files. For determination of differential miRNA expression, FES derived output data files were further analyzed using the GeneSpringGX (Version 12.6) analysis system (Agilent Technologies). Expression levels were log2 transformed and given in light units. If a miRNA is absent in the investigated sample the light units are automatically set by the FES to 0.1 light units, a value of −3.3 (log2 of 0.1) reflects therefore a non-expressed miRNA. Only those values were used with >100 lights units representing robustly expressed miRNAs. A heatmap was generated to visualize the expression levels of each miRNA (Spotfire, TIBCO Software Inc., Palo Alto, USA). The row and column dendrograms were clustered with the unweighted pair group method with arithmetic mean and Euclidean distance measure. Significant malaria-responsive and/or protective vaccination effects on miRNA expression were assessed using 2-way ANOVA. MicroRNAs with at least 2-fold deregulation and a *P* < 0.05 in any group-wise comparison were considered as to be differentially expressed. *P* values were corrected using Benjamini-Hochberg method for multiple testing (Benjamini and Hochberg, 1995). Raw data are publicly available at the EMBL-EBI Array Express repository (Array Express accession number: E-MTAB-5301).

### Quantitative real-time PCR

TaqMan® MicroRNA Reverse Transcription Kit (Applied Biosystems) and Megaplex™ RT Primers, Rodent Pool A and B (Applied Biosystems) were used to reverse transcribe miRNAs. Reactions were performed in triplicates using the following TaqMan® MicroRNA Assays (Applied Biosystems): let-7a-5p (assay ID: 00377), let-7c-5p (assay ID: 00379), miR-122-5p (assay ID: 002245), miR-142-3p (assay ID: 000464), miR-29b-3p (assay ID: 000413), miR-30c-5p (assay ID: 000419), miR-669n (assay ID: 197143_mat), miR-709 (assay ID: 001644), miR-92-3p (assay ID: 000430), miR-126-3p (assay ID: 002228). PCR reactions were performed with the TaqMan® gene expression master mix (Life Technologies) according manufacturer's protocol on a 7900HT real-time PCR System. The miRNA expression analysis was run on a SDS7900HT real time PCR system; raw Ct values were calculated using the SDS software v2.4 with automatic baseline and threshold settings. U6 snRNA (assay ID: 001973) was used for normalization. Fold change of expression was calculated using the comparative Ct method (2^−ΔΔct^) (Livak and Schmittgen, 2001). Data sets were analyzed for statistical significance using two-tailed unpaired heteroskedastic Student's *t*-test.

## Results

**Figure 1:**
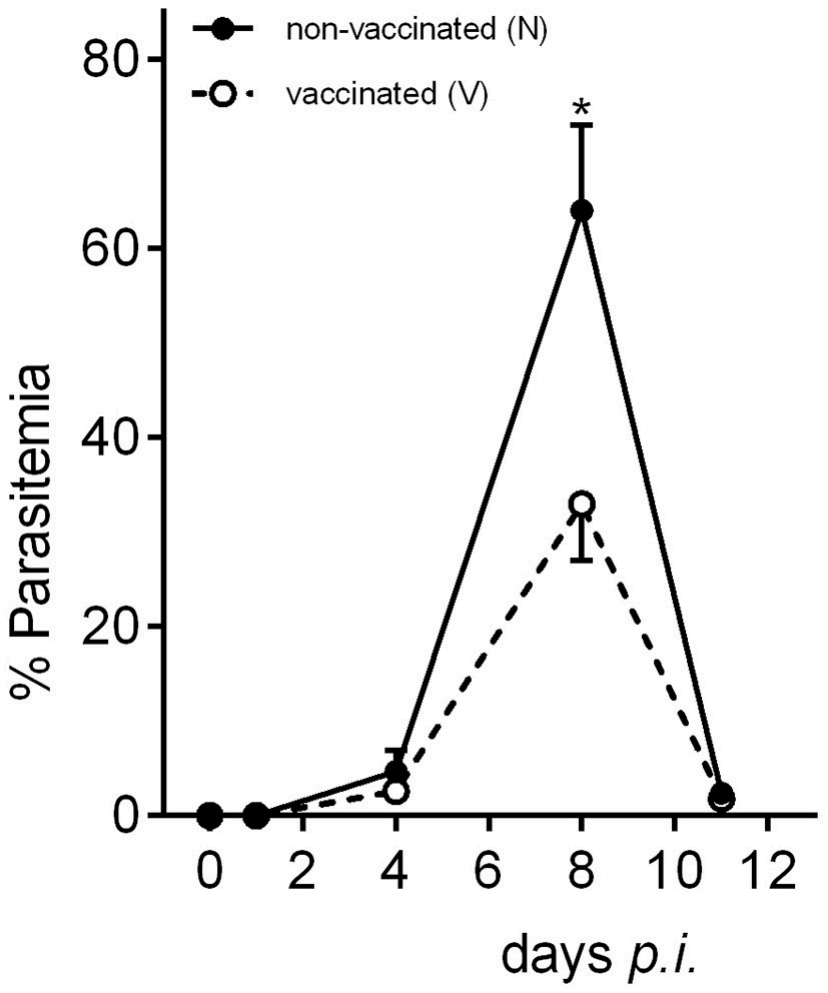
**Course of parasitemia during self-healing infections of ***P. chabaudi*** in vaccination-protected Balb/c mice and lethal infections in non-vaccinated mice**. Values represent means determined from three mice whose livers were taken to analyze miRNA expression. Bars represent half SD and star indicates significant difference (*p* < 0.01).

**Figure 2:**
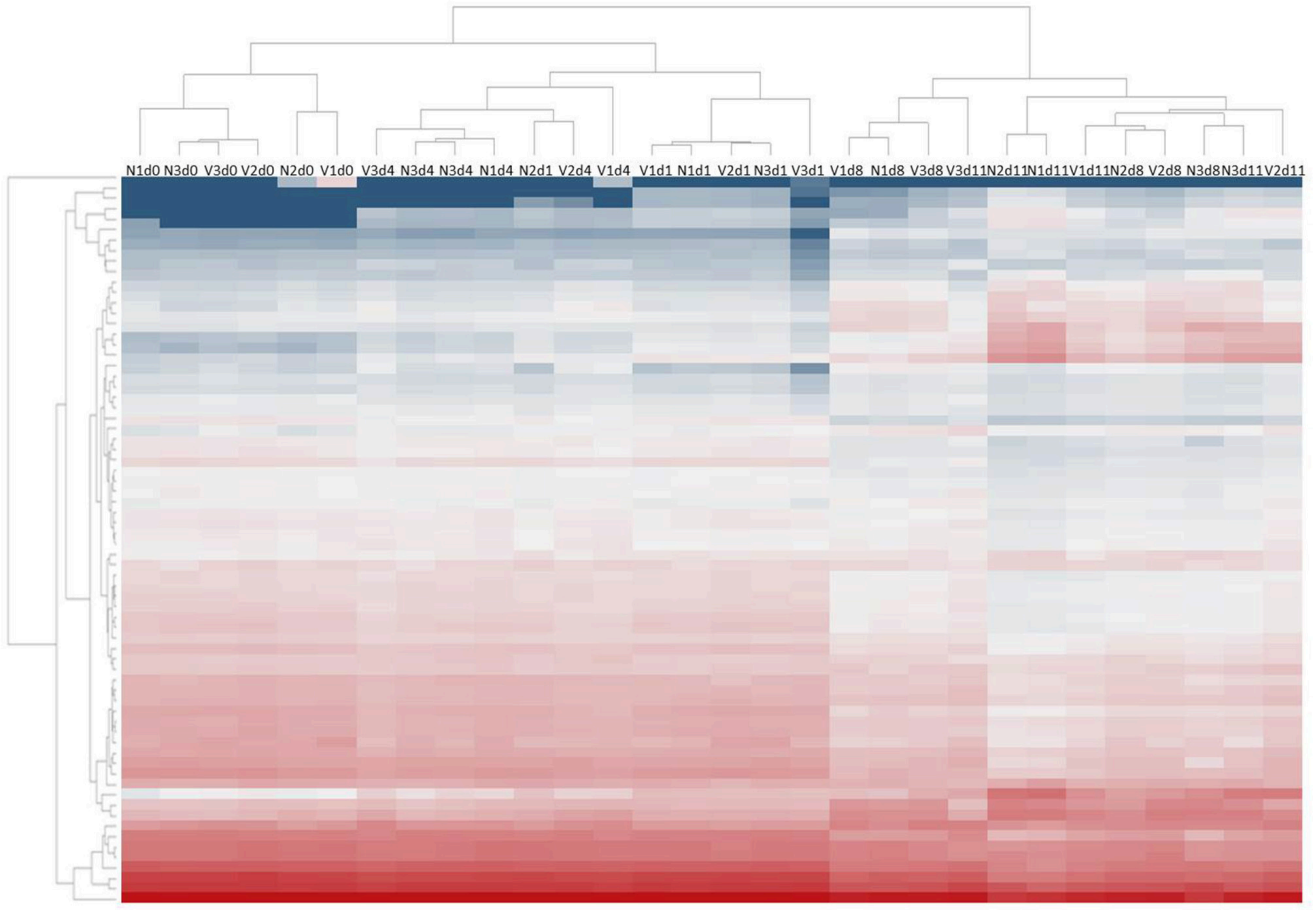
**Heatmap of expression levels of miRNAs in the liver of non-vaccinated (N) and vaccinated (V) mice infected with ***P. chabaudi*** on days 0, 1, 4, 8, and 11 ***p.i*****. Expression levels for each sample were hierarchically clustered. Log2 transformed expression levels range from −4 to 14 indicated by blue and red color, respectively.

To explore possible changes of hepatic miRNome by protective vaccination and/or subsequent challenge infections with *P. chabaudi* blood-stage malaria, microarrays were used to analyze miRNA expression in livers from vaccination-protected (V) and non-protected non-vaccinated mice (N) before infection on day 0 *p.i*. (Vd0, Nd0), and during different phases of the infections, i.e., at the beginning of infection on day 1 *p.i*. (Vd1, Nd1), during the mid-precrisis phase on day 4 *p.i*. (Vd4, Nd4), at peak parasitemia on day 8 *p.i*. (Vd8, Nd8), and toward the end of the crisis phase on day 11 *p.i*. (Vd11, Nd11). The parasitemia is about the same in self-healing infections of vaccination-protected mice and in lethal infections of non-protected mice (Figure 1). Only at peak parasitemia, there is a significant lower parasitemia in self-healing infections (Figure 1). Figure 2 shows the heat map of miRNA expression profiles during self-healing and lethal infections in vaccination-protected and non-protected mice, respectively. The profiles on day 0, 1, and 4 *p.i*. cluster separately from those on day 8 and 11 *p.i*., respectively, which are closer together.

Among the overall detectable 1247 miRNA species on a given array, 71 miRNA species are found to be robustly expressed (>100 light units) in the liver of both vaccinated and non-vaccinated mice. The most abundant expressions show the miRNAs miR-122-5p, miR-6366, miR-3963 and miR-5100, while relatively low expression is observed for the miRNAs miR-1196-5p, miR-468-3p and miR-669n. None of these miRNA species is affected by protective vaccination *per se*, evidenced as the expressions of the 71 miRNAs in vaccinated non-infected mice in comparison with the corresponding constitutive expressions in non-vaccinated mice. Upon infection with *P. chabaudi*, however, 65 miRNA species change their expression pattern, and 41 miRNA species out of the 65 malaria-responsive miRNAs significantly (*p* < 0.05) differ with respect to their expression levels in the liver between vaccinated and non-vaccinated mice. However, this difference is only evidenced toward the end of the crisis phase on day 11 *p.i*..

**Figure 3:**
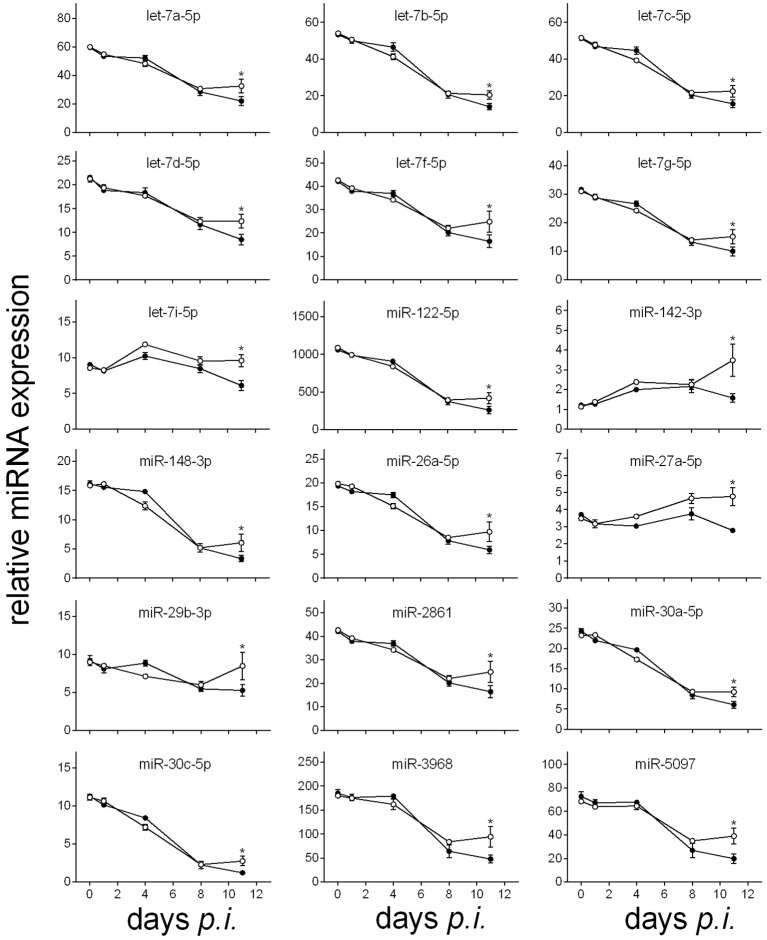
**Time course of relative miRNA expression with increased levels in the liver of vaccinated mice toward the end of the crisis phase of ***P. chabaudi*** infections in comparison to non-vaccinated mice**. Relative miRNA expression was normalized to the mean constitutive expression of the corresponding miRNAs. Open circles are values of vaccinated mice and filled circles are those of non-vaccinated mice. Values are given in mean ± SD. Significant differences are indicated by ^*^(*P* < 0.05).

**Figure 4:**
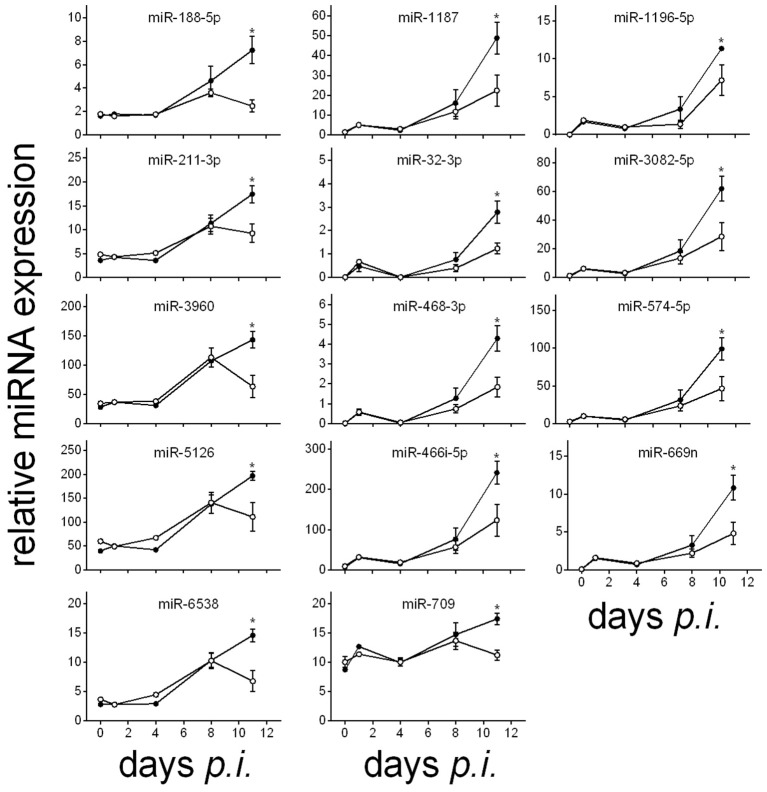
**Time course of relative miRNA expression of significantly decreased hepatic miRNA levels in vaccinated mice toward the end of the crisis phase of ***P. chabaudi*** infections compared to non-vaccinated mice**. Relative miRNA expression was normalized to the mean constitutive expression levels of the corresponding miRNAs. Open circles, vaccinated mice. Filled circles, non-vaccinated mice. Values represent means ± SD. Significant differences are indicated by ^*^(*P* < 0.05).

The lethal outcome of *P. chabaudi* malaria is associated with either increasing or decreasing expressions of miRNAs in the liver during the infections, reaching their minimal or maximal expression levels toward the end of the crisis phase on day 11 *p.i*. (cf. also Figures 3, 4). Decreasing expressions reveal the 18 miRNA species miR-122-5p, miR-142-3p, miR-148-3p, miR-26a-5p, miR-27a-5p, miR-29b-3p, miR-2861, miR30a/c-5p, miR-3968, miR-5097, and seven members of the let miRNA family (Figure 3), whereas increasing expressions occur for the 14 miRNAs miR-188-5p, miR1187, miR-1196-5p, miR-211-3p, miR-32-3p, miR-3082-5p, miR-3960, miR-466i-5p, miR-468-3p, miR-574-5p, miR-669n, miR-709, mir-5126, and miR-6538 (Figure 4). In vaccination-induced self-healing infections, however, the decreasing expression of the 18 miRNAs is stopped or even slightly turned to be up-regulated only by the end of crisis (Figure 3), whereas the increasing expression of the 14 miRNA species is impaired or even turned down (Figure 4). The up- or down-regulated expressions amount to more than 50% at the end of the crisis phase between vaccination-induced self-healing infections and lethal infections in non-vaccinated mice.

Tables 1, 2 summarize the annotated functions of the 18 and 14 miRNA species, whose expression is either up- or down-regulated in the liver of vaccination-protected mice toward the end of the crisis phase of *P. chabaudi* infections in relation to non-vaccinated mice, respectively. Remarkably, all the seven up-regulated members of the let-7 miRNA family are known to be involved in liver regeneration.

**Table 1:** **Increased hepatic miRNA expression in vaccinated mice infected with ***P. chabaudi*** on day 11 ***p.i***. (Vd11) compared to non-vaccinated mice infected with ***P. chabaudi*** on day 11 ***p.i***. (Nd11)**.

**miRNA name**	**% increase Vd11 vs. Nd11**	**Function**	**PMID**
let-7a	+50	Up-regulated during early phase of liver regeneration	21574170, 18812516
let-7b	+50	Up-regulated during early phase of liver regeneration	21574170, 18812516
let-7c	+50	Up-regulated during early phase of liver regeneration	21574170, 18812516
let-7d	+51	Up-regulated during early phase of liver regeneration	21574170, 18812516
let-7f	+51	Up-regulated during early phase of liver regeneration	21574170, 18812516
let-7g	+51	Up-regulated during early phase of liver regeneration	21574170, 18812516
let-7i	+57	Up-regulated during early phase of liver regeneration	21574170, 18812516
miR-122-5p	+60	Abundantly expressed in liver; involved in cholesterol biosynthesis pathway; binds to the HCV genome and enhances viral translation and replication	16258535,25574453, 16141076
miR-142-3p	+121	Orchestrates network of actin cytoskeleton during megakaryopiesis	24859754
miR-148a-3p	+81	miR-148a-3p downregulates Met/Snail signaling pathway and thus inhibits the epithelial to mesenchymal transition (EMT)	23532995
miR-26a-5p	+63	Involved in liver regeneration and hepatocyte proliferation; miR-26a expression reduced M-CSF expression and recruitment of macrophages in HCC	26818545, 26021873
miR-27a-3p	+71	Involved in lung fibrosis	26600197
miR-29b-3p	+61	Members of the miR-29 family are downregulated in HSCs in response to TNF and TGF-β signaling and suppress the transcription of ECM genes like collagen-1α1	20890893
miR-2861	+52	Important physiological role in osteoblast differentiation and contributes to osteoporosis via its effect on osteoblasts	19920351
miR-30a-5p	+52	Members of a network of miRNAs modifying the TGF-β-dependent regulation of extracellular matrix-related genes in HSCs in the manifestation and resolution of liver fibrosis	26120970
miR-30c-5p	+127	Members of a network of miRNAs modifying the TGF-β-dependent regulation of extracellular matrix-related genes in HSCs in the manifestation and resolution of liver fibrosis	26120970
miR-3968	+96	Unknown	
miR-5097	+98	Unknown	

**Table 2:** **Decreased hepatic miRNA expression in vaccinated mice infected with ***P. chabaudi*** on day 11 ***p.i***. (Vd11) compared to non-vaccinated mice infected with ***P. chabaudi*** on day 11 ***p.i***. (Nd11)**.

**miRNA name**	**% Increase Vd11 vs. Nd11**	**Function**	**PMID**
miR-188-5p	−65	Acts as a tumor suppressor in prostate caner	25714029
miR-1187	−54	Involved in hepatocyte apoptosis	22266786
miR-1196-5p	−37	Unknown	
miR-211-3p	−55	lncRNA-uc002kmd.1 regulates CD44 as a molecular decoy for miR211-3p	26974151
miR-32-3p	−54	Acts a a tumor suppressor in NSCLC	26229485
miR-3082-5p	−55	Unknown	
miR-3960	−56	miR-3960 regulated cellular growth and proliferation through a regulatory feedback loop with miR-2861, respnse to oxidative stress	21324897, 26539117
miR-466i-5p	−49	Unknown	
miR-468-3p	−57	Unknown	
miR-574-5p	−53	Oncogene in various cancer types, incl. SCLC	26587830
miR-669n	−55	Involved in control of LPS-induced macrophage activation	26807181
miR-709	−35	miR-709 may positively regulate invasion and metastasis of HCC through targeting GPC5	25818666
miR-5126	−43	Unknown	
miR-6538	−53	Unknown	

**Figure 5:**
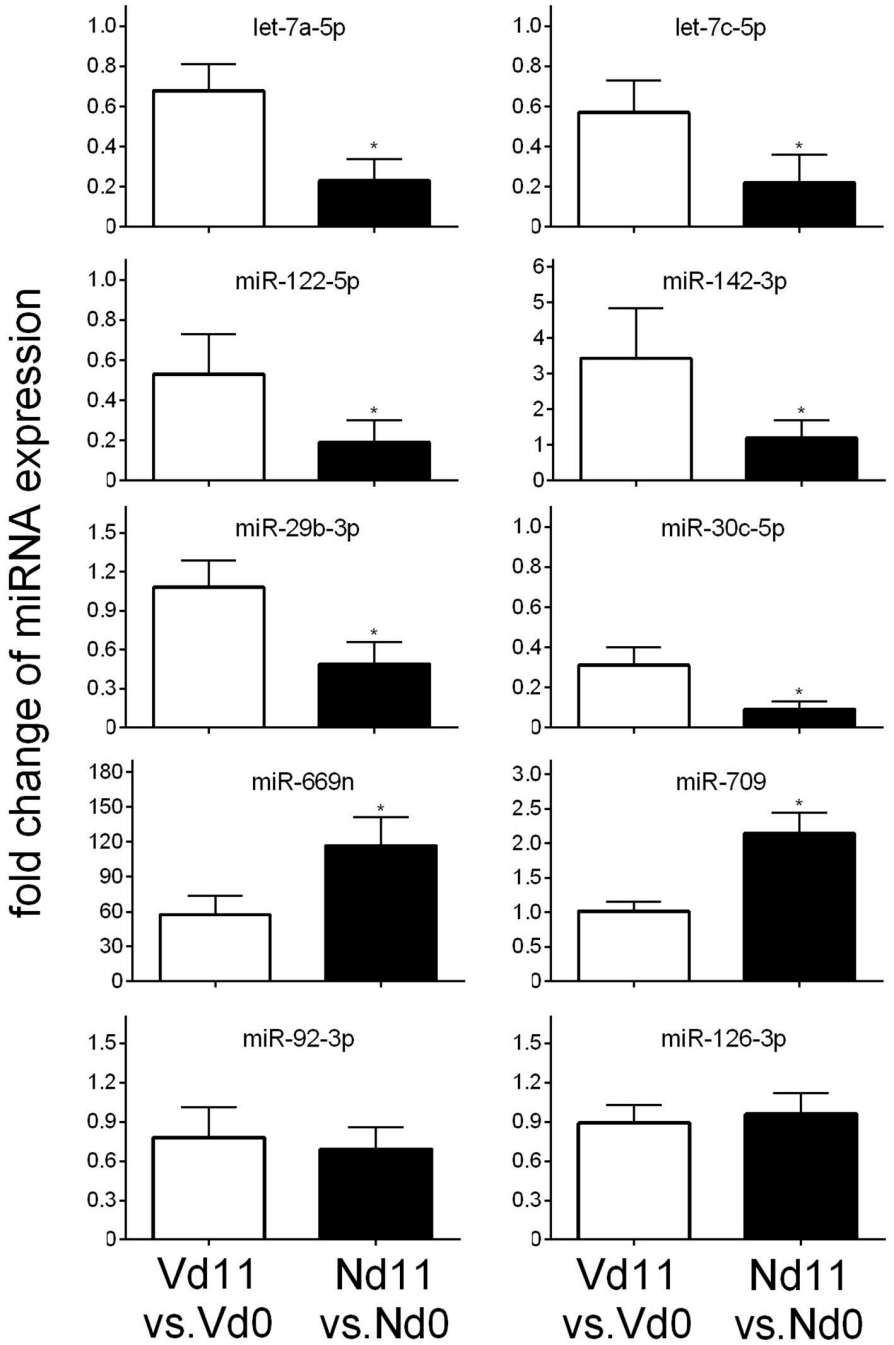
**Quantitative PCR of different miRNA species in the liver of vaccination-protected Balb/c mice infected with ***P. chabaudi*** on day 11 p.i. (Vd11) in relation to expression on day 0 p.i. (Vd0)**. Corresponding analyses for non-vaccinated mice (Nd11 vs. Nd0). Bars indicate half SD, (^*^) significant differences (*p* < 0.01).

To further substantiate these differences in miRNA expression toward the end of the crisis phase, we have also performed quantitative PCR of arbitrarily selected miRNAs. Figure 5 shows that the miRNAs let-7a-5p, let-7c-5p, miR-122-5p, miR-142-3p, miR-29b-3p, and miR-30c-5p are up-regulated and the miRNA species miR-669n and miR-709 are down-regulated in livers of vaccination-induced self-healing infections on day 11 *p.i*. in comparison to lethal infections in non-vaccinated mice, thus confirming our microarray analyses. By contrast, the expressions of the malaria-responsive miR-92-3p and miR-126-3p are not significantly affected by infections, as it was also found by microarrays.

## Discussion

This is the first study providing evidence that protective vaccination against blood-stage malaria of *P. chabaudi* leads to changes in the expression of distinct miRNA species in the liver during subsequent homologous challenge infections. These changes, however, are evidenced only toward the end of the crisis phase on day 11 *p.i*. in relation to lethal infections in non-vaccinated mice. Lethal malaria induces increasing expressions of 18 miRNA species and decreasing expressions of 14 miRNA species during the precrisis phase reaching highest and lowest expression levels toward the end of the crisis phase. In vaccinated mice, however, the course of the decreasing and increasing expressions of the 18 and 14 miRNA species, respectively, is apparently stopped at peak parasitemia and even partly reversed during crisis, resulting in changes by more than 50 % in relation to non-vaccineted mice toward the end of the crisis phase. This suggests that vaccination-induced self-healing infections of *P. chabaudi* blood-stage malaria are associated with a remodeling of miRNA-controlled processes in the liver of Balb/c mice, which otherwise would lead to a lethal outcome of the infections during crisis.

Such a remodeling is presumably associated with an accelerated liver regeneration. Indeed, seven members of the let-7 miRNA family, found here to be up-regulated in the liver of vaccination-protected mice during crisis, have been all described to be involved in liver regeneration by targeting mRNAs of dicer and trbp (Forman et al., 2008; Shu et al., 2011). Also, the mir-26a up-regulated during crisis is known to be involved in liver regeneration and hepatocyte proliferation (Zhou et al., 2016). Moreover, the down-regulated expression levels of miR-1187 may reflect diminished hepatocyte apoptosis through targeting casp-8 (Yu et al., 2012). Furthermore, the down-regulated levels of miR-669n during crisis may conceivably contribute to accelerated liver regeneration by dampening malaria-induced Kupffer cell activation (Long et al., 2015). All these data support the view that liver regeneration may be accelerated in vaccination-protected mice during the crisis phase of self-healing infections of otherwise lethal *P. chabaudi* malaria. Accelerated liver regeneration in vaccinated mice is presumably not a direct effect of vaccination, but rather it may be associated with the process of self-healing, i.e., it may be an indirect consequence of the vaccination-induced conversion of lethal to self-healing infections of *P. chabaudi*.

Accelerated liver regeneration may contribute to overcome dysfunctions of the liver, presumably due to injuries induced by the *P. chabaudi* blood-stage infections. Indeed, the acute phase of *P. chabaudi* malaria has been described to be associated with pathological damages and heavy injuries in the liver with distant effects on other organs, as e.g., hepatoencephalopathy (Wunderlich et al., 2005; Delic et al., 2010). Even human patients suffering from malaria with *P. falciparum* and *P. vivax* have been shown to be associated with massive dys-functions of the liver (Ananad et al., 1992; Kochar et al., 2003; Nautyal et al., 2005; Rupani and Amarapurkar, 2009). Moreover, it is known that pathogenesis and diseases of the liver are in general associated with dys-regulated expressions of diverse miRNAs (Szabo and Bala, 2013; Chen and Verfaillie, 2014; Bandiera et al., 2015; Murakami and Kawada, 2016). For instance, injuries of the liver in HIV/HCV patients suffering from necroinflammation and portal hypertension have been recently reported to cause elevated levels of the miR-122, the most abundant miRNA species in the liver (Jansen et al., 2015). Our data, however, reveal continuously decreasing levels of miR-122-5p in the liver of both vaccinated and non-vaccinated mice during the precrisis phase of self-healing and lethal *P. chabaudi* infections, respectively. Only during the crisis phase, vaccination-protected mice exhibit elevated levels of miR-122-5p, whereas the corresponding levels further decrease in non-vaccinated mice with lethal infections. Similar courses of miRNA expression levels are here found for miR-30a known to be involved in manifestation and resolution of liver fibrosis (Roy et al., 2015), for miR-27a described to be involved in fibrosis (Cui et al., 2016), and for mir-29b known to suppress transcription of genes encoding for extracellular matrix proteins (cf. Table 1) (Roderburg et al., 2011; Lambrecht et al., 2015; Kitano and Bloomston, 2016). Thus, the dys-regulations of these different miRNA species may presumably due to the liver injuries induced by *P. chabaudi* blood-stage malaria occurring during precrisis in both vaccination-protected and non-vaccinated mice. However, the up-regulated levels of mir-122-5p, miR-30a, mir-27a, and miR-29, observed in vaccination-protected mice during crisis, may contribute to the accelerated liver regeneration suggested to occur in these mice.

Moreover, the present study shows that the expression level of mir-142-3p is significantly up-regulated during crisis by more than 100% in the liver of vaccinated mice as compared with non-vaccinated mice, i.e., this percental difference during crisis is by far higher than those found for all other malaria-responsive miRNA species in the liver. Recent evidence indicates that the miRNA-142 locus is important for the regulation of macrophage-related processes, as e.g., macrophage and dendritic cell differentiation (Fordham et al., 2015), regulation of cell migration (Kim et al., 2015), control of profibrogenic macrophage program (Su et al., 2015), role in colony-stimulating factor 1-induced monocyte differentiation into macrophages (Lagrange et al., 2013), prevention of macrophage differentiation during cancer-induced myelopoiesis (Sonda et al., 2013). The relevance of these data for the liver of vaccinated mice remains to be shown. Currently, however, the most unequivocal evidence demonstrates that mir-142-3p is critical for megakaryopoiesis (Chapnik et al., 2014). The miRNA-142-3p stringently controls specific cytoskeletal rearrangements required for maturation and function of megakaryocytes, and genetic deletion of miR-142-3p results in impaired megakaryocyte ablation, cytoskeletal dys-integrity, abnormal proplatelet formation and thrombocytopenia (Chapnik et al., 2014). In accordance, thrombocytopenia also occurs in experimental malaria including *P. chabaudi* malaria (Watier et al., 1992; Piguet et al., 2000, 2002; Gramaglia et al., 2005) and even represents a severe complication in human malaria caused by *P. vivax* and *P. falciparum* (Ansari et al., 2009; Lacerda et al., 2011; Gill et al., 2013; Gupta et al., 2013). Thus, our data showing up-regulation of mir-142-3p may suggest that protective vaccination affects hepatic megakaryopoiesis induced by *P. chabaudi* blood-stage malaria during crisis.

On the other hand, however, there is evidence that miR-142-3p is also involved in the regulation of erythropoiesis (Muhseen and Abbood, 2014). Erythropoiesis in turn is tightly coupled with megakaryopoiesis. Both erythropoiesis and megakaryopoiesis are controlled by the two transcription factors GATA1 and GFI1b (Fatica et al., 2006; Crispino and Weiss, 2014). Moreover, both megakaryocytic and erythroid cells are derived from common myeloid precursors (Fatica et al., 2006), and there exist even bipotent progenitor stem cells generating both erythroid and magakaryoctic cells (Debili et al., 1996; Papayannopoulou et al., 1996; Papayannopoulou and Kaushansky, 2016). However, there is also information available that erythropoiesis is enhanced at the expense of megakaryopoiesis (Bianchi et al., 2015). Under identical experimental conditions as described here, Al-Quraishy et al. (2016) have recently found that protective vaccination augments *P. chabaudi*-induced extramedullary hepatic erythropoiesis. It appears therefore plausible to assume that the up-regulation of mir-142-3p observed here during crisis is involved in enhanced erythropoiesis at the expense of megakaryopoiesis in the liver of vaccination-induced self-healing infections of *P. chabaudi* malaria (Al-Quraishy et al., 2016).

Collectively, our data suggest that vaccination-induced self-healing and survival of otherwise lethal blood-stage infections of *P. chabaudi* malaria activates epigenetic miRNA-regulated mechanisms in the liver, which accelerate liver regeneration and enhance hepatic erythropoiesis, possibly at the expense of megakaryopoiesis, during crisis of *P. chabaudi* blood-stage malaria.

## Author contributions

MD, SA, and FW designed the study; MD, AA, and DD carried out the experiments and analyzed the data. All authors wrote and revised the manuscript.

### Conflict of interest statement

The authors declare that the research was conducted in the absence of any commercial or financial relationships that could be construed as a potential conflict of interest.
